# Development of Innovative Composite Nanofiber: Enhancing Polyamide-6 with ε-Poly-L-Lysine for Medical and Protective Textiles

**DOI:** 10.3390/polym16142046

**Published:** 2024-07-17

**Authors:** Saloni Purandare, Rui Li, Chunhui Xiang, Guowen Song

**Affiliations:** Department of Apparel, Events, and Hospitality Management, Iowa State University of Science and Technology, Ames, IA 50011, USA; salonipp@iastate.edu (S.P.); ruili@iastate.edu (R.L.)

**Keywords:** ε-poly-l-lysine, polyamide, antimicrobial textiles, hydrophilic, nanofiber

## Abstract

Polyamide-6 (PA) is a popular textile polymer having desirable mechanical and thermal properties, chemical stability, and biocompatibility. However, PA nanofibers are prone to bacterial growth and user discomfort. ε-Poly-L-lysine (PL) is non-toxic, antimicrobial, and hydrophilic but lacks spinnability due to its low molecular weight. Given its similar backbone structure to PA, with an additional amino side chain, PL was integrated with PA to develop multifunctional nanofibers. This study explores a simple, scalable method by which to obtain PL nanofibers by utilizing the structurally similar PA as the base. The goal was to enhance the functionality of PA by addressing its drawbacks. The study demonstrates spinnability of varying concentrations of PL with base PA while exploring compositions with higher PL concentrations than previously reported. Electrospinning parameters were studied to optimize the nanofiber properties. The effects of PL addition on morphology, hydrophilicity, thermal stability, mechanical performance, and long-term antimicrobial activity of nanofibers were evaluated. The maximum spinnable concentration of PL in PA-based nanofibers resulted in super hydrophilicity (0° static water contact angle within 10 s), increased tensile strength (1.02 MPa from 0.36 MPa of control), and efficient antimicrobial properties with long-term stability. These enhanced characteristics hold promise for the composite nanofiber’s application in medical and protective textiles.

## 1. Introduction

Nanofibers are fibers with a diameter less than 1000 nm. Nanofibers have been gaining increasing attention due to advantageous characteristics such as high surface-to-volume ratio, low-pressure drop, good interconnectivity of voids, and controllable connectivity and morphology. Nanofibers find applications in a wide range of areas owing to their advantages, particularly in medical and protective textiles [1]. Electrospinning is a commonly used method by which to spin nanofibers wherein a viscoelastic spinning solution is fed at a continuous rate through a needle, and the needle is supplied with high voltage. The electrostatic forces overcome the surface tension of the drop at needle tip to form a jet. The jet stretches and solidifies into nanofiber on its way to a grounded collector [2]. The popular use of the electrospinning technique is mainly due to its simple, cost-effective setup and ability to manipulate the surface features and morphology of nanofibers [3,4].

Functional textiles refer to a category of textile products, specially engineered for a predetermined functionality above body covering and aesthetics. The vast area of functional textiles includes several categories, such as, but not limited to, protective, medical, sports, and electronic textiles [5]. Functional textile research has established several innovative solutions that enhance the quality of life of the user. However, the optimization of functional textiles is an on-going journey, with challenges in capturing real-use scenarios along with factors such as the scalability, durability, reusability, and non-toxicity of the textile [6]. One of the categories of functional textiles is antimicrobial textiles. The antibiotic resistance of bacterial strains and unavoidable bacterial contamination despite rigorous hygiene protocols calls for antimicrobial surfaces in medical and protective settings [7,8]. Antimicrobial function in textiles prevents microorganisms from reaching the wearer and vice versa; therefore, it remains essential for a healthy environment, especially in a medical setting. The antimicrobial function also allows for the material to be self-decontaminating, ensuring the textiles’ usability throughout its shelf time [9,10]. Leaching and synthetic antimicrobial agents exhibit toxicity towards the wearer, therefore highlighting the importance of naturally occurring non-toxic antimicrobial agents [10,11].

ε-poly-L-lysine (PL) is a cationic homopolyamide naturally produced by the filamentous bacterium Streptomyces albulus [12,13]. The naturally occurring homopolyamide has an antimicrobial function owing to the cationic α-amino group. PL has been found to exhibit an antimicrobial function through the destruction of microbial growth via electrostatic adsorption towards a wide range of Gram-positive and Gram-negative bacteria [12,13,14]. Either naturally or bio-synthetically, PL is commonly available at low molecular weights. The available low molecular weight (3600–4300 Da) has been established for effective antimicrobial function due to the presence of 25–30 L-lysine residues exhibiting cationic action against microorganisms. However, the low molecular weight limits the spinnability of PL into a material [12,14,15]. Thus, a nanocomposite of PL, only in small concentrations with carrier polymers such as chitosan [16,17], gelatin [18,19], and poly(hydroxybutyrate) [20], is being increasingly explored for a wide range of applications such as food preservation, food packaging, wound dressing, and drug carrying. Studies have exhibited an improved bactericidal effect with increased bacterial damage upon increasing the concentration of PL [19,21]. The results of several analyzed studies also support improved antibacterial properties with increased PL concentration [16,17,22]. Further, cationic polymers, such as PL, experience a proliferation of bacteria on the surface once the surface is sufficiently covered with microorganisms due to the exhaustion of cations [7,23,24]. A fiber system with an increased percentage of active antimicrobial sites can be expected to have efficient and stable contact-induced antimicrobial function. Along with improved antimicrobial efficiency, studies have also shown improved hydrophilicity with increased PL concentration [16,17,19]. Therefore, there is an existing gap in the research concerning a fiber system with a high concentration of PL, possibly via copolymerization or the utilization of a carrier system with a polymer of similar backbone structure such as polycaprolactam [15].

Polyamide is a polymer largely utilized in textiles owing to its good mechanical and thermal properties, chemical stability, and biocompatibility. Polyamide is easily spinnable into homogeneous uniform nanofibers that are biocompatible but non-degradable, which allows for application of a permanent nature and encourages reusability. Therefore, polyamide is commonly found in functional textiles. Polyamide nanofibers are largely being explored for air filtration due to their ability to form fine homogenous nanofibers with small pore size [25,26]. Polyamide is also increasingly being explored for food packaging and medical textiles. A drawback associated with polyamide nanofibers is that they are a suitable carrier for bacterial growth. The drawback is addressed through the incorporation of an antimicrobial agent and morphological manipulation, avoiding non-homogeneous thick nanofibers [27,28,29].

Polyamide-6 (PA), also known as polycaprolactam, is formed via the ring-opening polymerization of hexamethylenediamine. The polymer has a chemical structure of (C6H11NO)n, with the lactam monomers held together by amide linkages. On the other hand, PL has a similar main backbone structure to PA, with the additional cationic amino side chains [30,31]. Thus, a combination of PL with PA can be expected to result in spinnable fibers with a backbone of PA, with the added cationic alpha-amino group providing an antimicrobial function [15]. PL has an inherent antimicrobial function, is hydrophilic, and has low solution viscosity [15]. Thus, the incorporation of PL to PA nanofibers, along with the incorporation of an antimicrobial function, is hypothesized to improve the characteristics of PA by forming uniform nanofibers with enhanced hydrophilicity. The non-toxic antimicrobial function will address the tendency of PA to be a carrier of bacterial growth [27,28,29]. Further, the improved hydrophilicity of PA can enhance user comfort while benefiting several forms of end-use, including the inner filter of a mask system, wound dressing, and sanitation [9,32,33]. This study thus explores a simple scalable method of obtaining PL nanofibers by utilizing the structurally similar PA as the base. Also, this study aims to enhance the utilization of PA polymer in medical and protective textiles by addressing its drawbacks of bacterial growth and user discomfort.

## 2. Materials and Methods

### 2.1. Materials

Polyamide-6 (PA) (Mw = ~10,000 Da) and formic acid (88%, Macron Fine Chemicals) were purchased from Sigma-Aldrich (St. Louis, MO, USA). PL (Mw = ~3500–4700 Da) was purchased from Zhengzhou Bainafo Bioengineering Co., Ltd. (Henan, China). *Escherichia coli* K–12 bacterial culture and nutrient agar was purchased from Carolina Biological Supply Company (Burlington, NC, USA).

### 2.2. Preparation of Electrospinning Solution

Spinning solution was prepared by dissolving PA along with varying concentrations of PL in formic acid (88% concentration) under constant stirring overnight with a Burrell wrist-action^®^ shaker, model-75 (Burrell Scientific LLC, Pittsburgh, PA, USA), to obtain a homogeneous solution. Formic acid was chosen as it allows for an environmentally friendly process over other toxic solvents like dimethylformamide (DMF). Also, smooth PA fibers are expected with the organic solvent formic acid in comparison with other solvents like m-cresol or sulphuric acid, even at a low polymer concentration [25,34]. The spinning solution compositions and sample codes are described in Table 1.

### 2.3. Electrospinning

Nanofiber membranes were developed using a single-needle electrospinning setup. The solution was continuously fed at a fixed rate using a syringe pump (Harvard Apparatus, Holliston, MA, USA). The distance between the needle tip and the grounded collector was set, and a fixed high voltage was applied to the needle tip by a DC power supply instrument (Gamma High Voltage Research, Ormond Beach, FL, USA). Table 2 specifies the spinning parameters used for all the compositions.

### 2.4. Morphological Analysis

Morphological analysis of the electrospun nanofibers for their diameter and surface features was performed using a field emission scanning electron microscope (FESEM) (FEI Quanta 250, ThermoFisher Scientific, Waltham, MA, USA). Test samples were kept in a vacuum overnight to evaporate any residual solvent prior to imaging. Image J software (National Institute of Health, Bethesda, MD, USA) was used to determine the diameter of the nanofibers. The average diameters were determined by measuring diameters of 50 representative fibers.

### 2.5. Fourier Transform Infrared (FTIR) Spectroscopy

The functional groups and presence of PL in developed nanofiber membranes were determined using an FTIR spectrometer (Perkin-Elmer Frontier FTIR, Waltham, MA, USA). The FTIR spectra were obtained in the range of 500–4000 cm^−1^ with 4 cm^−1^ wavenumber resolution, and each measurement consisted of 32 scans.

### 2.6. Water Contact Angle

The static water contact angle was measured as per the ASTM-D7334-08 sessile drop method [35]. The testing was conducted using a video-based drop shape analyzer (OCA 25, Data Physics GmbH, Regensburg, Germany), and the contact angles were measured with the built-in SCA20 software (V.4.5.20). For each sample, the contact angle measurement was repeated five times on separate membranes, and an average of the readings is reported.

### 2.7. Water Absorption Test

The water absorption capacity was measured through the swelling ratio. The initial dry weight of 1 cm × 1 cm sample was calculated (w1). Samples were then submerged in excess distilled water for 24 h, followed by the measurement of their swollen weights (w2). The swelling ratio of each sample was then calculated in triplicate on separate membranes using the following Equation (1) [36,37]:

(1) $$\mathrm{Swelling}\;\mathrm{ratio}\;(g/g)=\frac{w2-w1}{w1}.$$

### 2.8. Thermal Analysis

Thermal analysis was conducted using a differential scanning calorimeter (PerkinElmer DSC 4000, Shelton, CT, USA). Each sample (approximately 10 mg) was heated to 400 °C at a heating rate of 20 °C min^−1^.

### 2.9. Mechanical Analysis

The tensile properties of the samples were tested as per ASTM-D882-09 using an Instron 5966 (Instron, Boston, MA, USA) tensile testing machine [38]. The samples were tested with a load cell of 250 N, with 4 cm between the clamps, and at a stretching rate set at 5 mm/min^−1^. Test specimens were prepared in dimensions of 4 × 1 inches and were conditioned at ambient conditions for a day prior to testing. Testing was conducted on three separate membranes for each sample, and an average of three readings is reported.

### 2.10. Antimicrobial Analysis

The antimicrobial testing was performed as per the AATCC-147 zone inhibition method [39]. Each sample was cut into circles of 1 cm diameter. Nutrient agar plates were prepared and seeded with 1 mL *E. coli* inoculums containing approximately 3 × 10^6^ colony forming units (CFU)/mL. The samples were placed on the plates and incubated for 24 h at 37 °C. Bacterial growth post-incubation for each sample was observed. Testing was conducted in triplicate on separate membranes for each composition.

### 2.11. Long Term Stability of Antimicrobial Property

To understand the ideal shelf life of the developed nanomembrane, the antimicrobial function was analyzed following AATCC-147 as described above at different time intervals (1 and 6 months) in triplicate. In between the testing, the nanofiber membranes were stored in plastic bags at room temperature.

### 2.12. Statistical Analysis

In this study, the results were analyzed using SPSS software (version 29.0; IBM Corp., Armonk, NY, USA) using one-way analysis of variance (ANOVA) and Tukey statistical tests. All results are expressed as mean ± standard deviation, and a *p*-value < 0.05 was considered significant.

## 3. Results

### 3.1. Electrospinning of PA/PL Nanofiber Membranes

**Figure 1:**
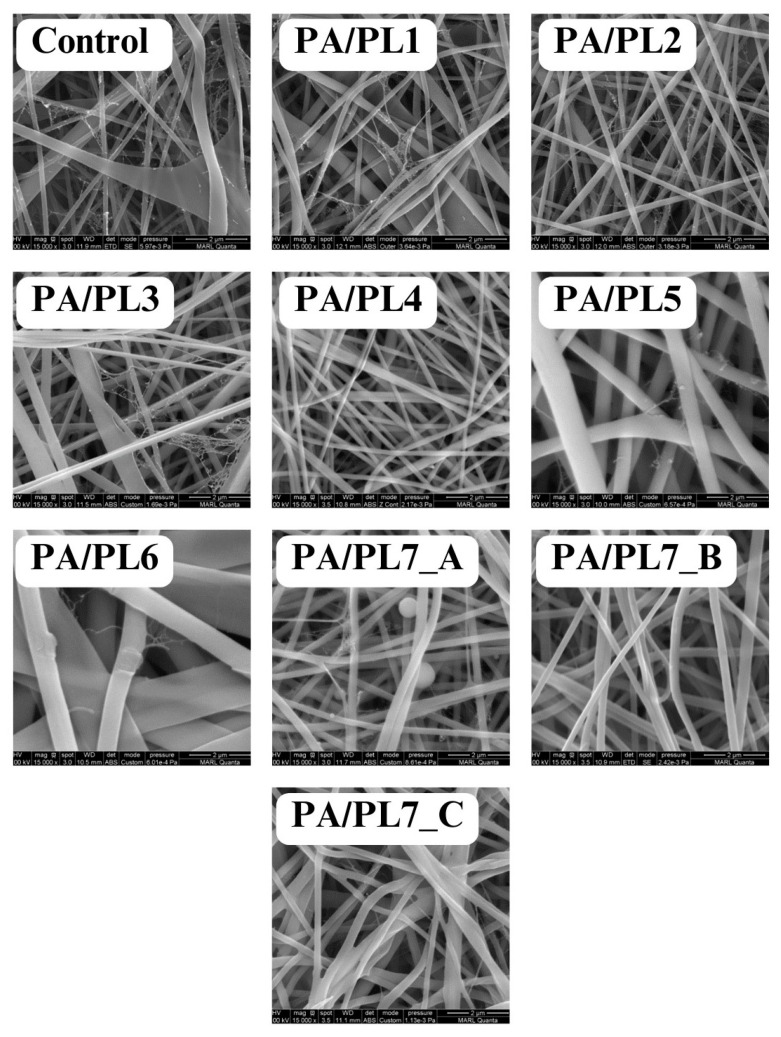
FESEM images of nanofiber membranes at 15,000×.

Solution properties and spinning parameters are crucial for the successful execution of the electrospinning process. A delicate balance between the influential parameters is required to obtain smooth uniform nanofibers. For the successful spinning of PA into smooth uniform nanofibers, spinning parameters were set for the control sample (see Table 2). The parameters were further adjusted to ensure spinnability with increasing concentration of PL. Compositions PA/PL1 to PA/PL5 resulted in defect free nanofibers, thus validating the spinning parameters used for the control sample. While PA/PA6 resulted in a flat nanoribbon structure with non-uniform diameter distribution (see Figure 1). The nanoribbon structure of this composition could be a result of increased solution viscosity due to the large amount of PL addition resulting in high polymer content in the system. The increased spinning solution viscosity results in thick jets during electrospinning. The dissipation of the solvent during the jet’s flight from the spinneret to the collector is compromised due to the jet’s excessive thickness. The dissipation of solvent molecules from the jet core becomes difficult, resulting in a polymer skin on the jet surface. This jet collapses upon reaching the collector due to the solvent-rich interior forming the nanoribbon structure [2,40,41]. Thus, PA/PL5 was considered to be the maximum possible concentration of PL that can be added to the control spinning solution for set spinning parameters.

Further increment of PL required reduction in the base polymer concentration. Thus, for PA/PL7, the concentration of PA was reduced to 15% (*w*/*v* of formic acid) from the original 30% (*w*/*v* of formic acid). PA/PL7 was spun with varying parameters to determine suitable spinning parameters for the reduced PA and increased PL concentration (see Table 2). The voltage applied in electrospinning should be consistent with the viscosity to ensure repulsive forces to overcome the surface tension of the solution for the formation of Taylor’s cone. Excessively high voltage can result in defects due to high jet instability and velocity [2,42,43]. The reduced base polymer content in the sample PA/PL7 is expected to reduce solution viscosity. Thus, PA/PL7_A was spun at a reduced voltage of 18 KV compared to the original 20 KV voltage. Despite the reduced voltage, unspun beads were observed during the morphological analysis of PA/PL7_A (see Figure 1). The unspun beads could be a result of jet breakage due to high jet velocity facilitated by excess voltage. Thus, a further decrement in voltage to 16 KV was applied to PA/PL7_B and PA/PL7_C. Along with decreasing voltage, the spinning distance was increased in PA/PL7_C as beads could also arise from insufficient stretching of the jet. The decreased voltage in PA/PL7_B resulted in nanofibers; however, a few fibers exhibited a flat-ribbon-like morphology, while the combination of decreased voltage with increased spinning distance in PA/PL7_C facilitated increased nanoribbon morphology (see Figure 1). The nanoribbon structure could be attributed to the decreased voltage ejecting thick jets. The thick jets during the flight time develop an outer polymer skin and a solvent-rich interior, which collapses into ribbon morphology. The increased distance seemed to facilitate the ribbon morphology due to the increased flight time assisting the outer layer of the thick jet in its solidification, thus preventing the dissipation of solvent from the core [40]. Overall, the PA/PL7 series resulted in poor yield owing to the reduced base polymer concentration.

### 3.2. Morphological Analysis

**Figure 2:**
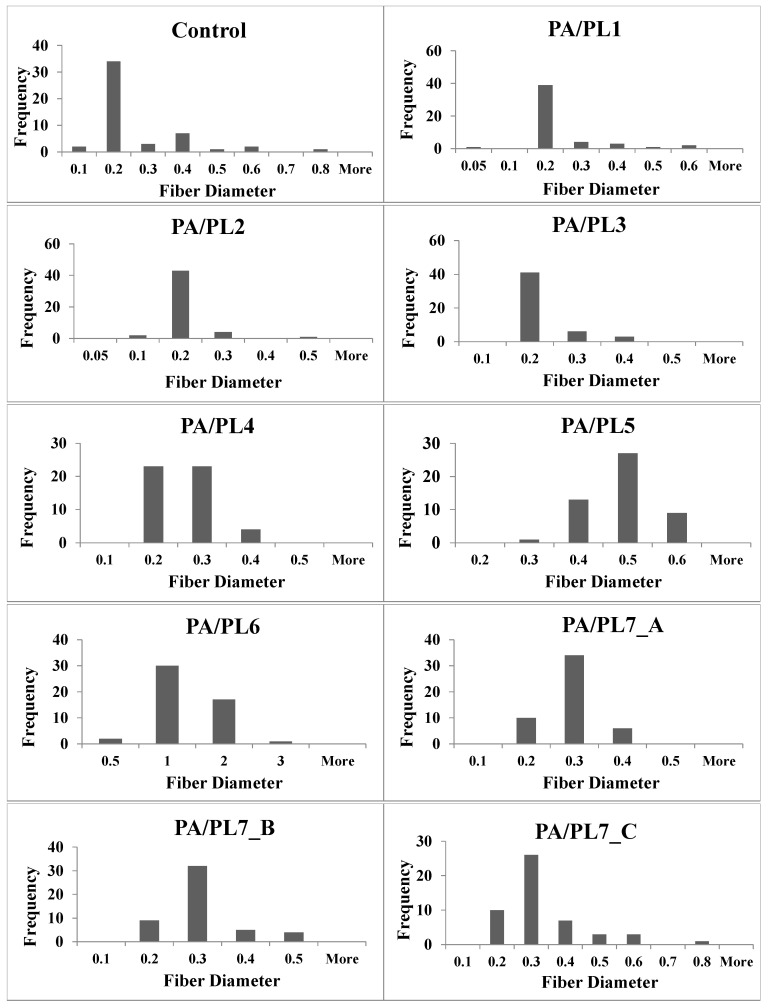
Fiber diameter distribution.

The effect of PL addition on the morphology and diameter of nanofibers was analyzed using FESEM (see Figure 1). Table 3 summarizes the effect of PL addition on the diameter of the nanofibers. Figure 2 shows the fiber diameter distribution for each sample. A trend observed was a decrease in diameter from the control sample for PA/PL1 to PA/PL4. PL is of a polycationic nature and has hydration capacity, resulting in reduced spinning solution viscosity and increased conductivity. This allows for greater stretching of the spinning jet during electrospinning, thus explaining the thinner nanofibers [18,44,45]. Although PL addition to base PA polymer reduced the diameter of the nanofibers, the addition of PL beyond a certain amount—in this case, PA/PL5 and further—increases the diameter. This is due to the increased polymer content with respect to the ratio of the solvent resulting in increased solution viscosity.

Nanoweb structures were observed in all the membranes. The formation of spider-net structures between the polyamide nanofibers upon the use of formic acid as a solvent is because of the interaction between the amide group of PA and the ionic species in formic acid [25]. These structures are expected to improve applicability in areas such as filtration.

Based on the morphological analysis, defective structures, which were elaborated in the previous section, were excluded from further testing. Thus, the test samples for further characterization constitute defect-free homogenous nanofibers, namely, control, PA/PL1, PA/PL2, PA/PL3, PA/PL4, and PA/PL5.

### 3.3. Fourier Transform Infrared (FTIR) Spectroscopy

**Figure 3:**
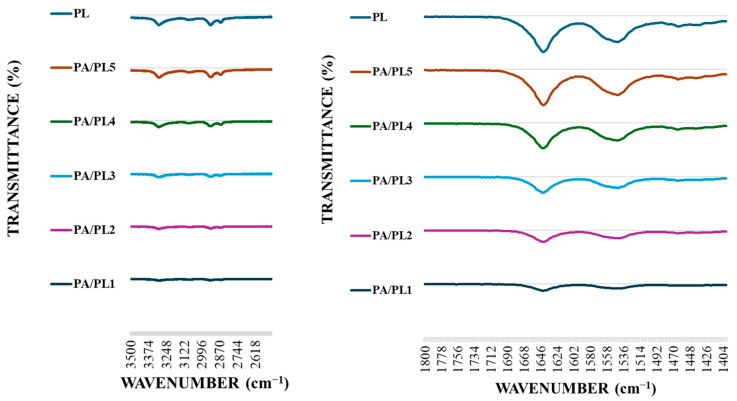
FTIR spectroscopy of PL and PA/PL nanofiber membranes.

The FTIR analysis demonstrated characteristic peaks of both PL and PA, confirming their presence in the nanofiber membranes (see Figure 3). Samples exhibited characteristic bands of PL with peaks at 1661 cm^−1^ and 1555 cm^−1^, indicating C=O and N-H_2_ stretching vibration, respectively [17,18]. These peaks indicate the presence of PL. The absorption intensity of characteristic peaks of PL became stronger with the increasing concentration of PL, indicating the successful incorporation of a high concentration of PL in the system [46]. Peaks observed at ~3300 cm^−1^ can be attributed to hydrogen-bonded N-H or O-H stretching vibration. The absorption intensity of these peaks also became stronger with increasing concentration of PL in the base PA, suggesting hydrogen bonding between the polymer chains during electrospinning [47]. Additionally, all the PA/PL samples exhibited peaks at ~2900 cm^−1^ and 2935 cm^−1^. These peaks signify C-H_2_ stretching of polyamide [48]. Therefore, the FTIR analysis indicates the successful incorporation of PL to base PA and exhibits the possibility of interaction between the polymers.

### 3.4. Analysis of Hydrophilic Behavior

#### 3.4.1. Water Contact Angle

**Figure 4:**
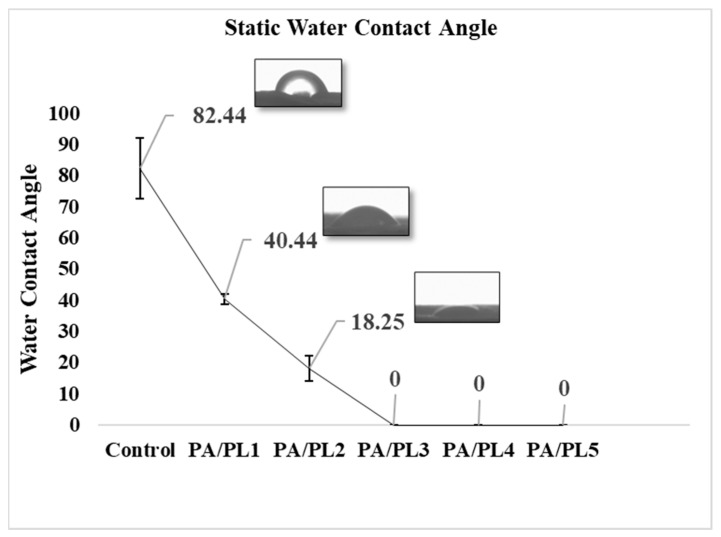
Static water contact angle of nanofiber membranes.

Static water contact angles of the samples were measured through the sessile-drop method using a video-based drop shape analyzer. As seen in Table 4 and Figure 4, the contact angle significantly decreases with the addition of PL. The decreased contact angle indicates improved hydrophilicity. The contact angles for the compositions PA/PL3, PA/PL4, and PA/PL5 were zero within the first 10 s of testing, indicating highly hydrophilic behavior. The improved hydrophilicity upon PL addition could account for its inherent hydrophilic nature due to the large amino and amido groups [17,19,33].

#### 3.4.2. Water Absorption Test

**Figure 5:**
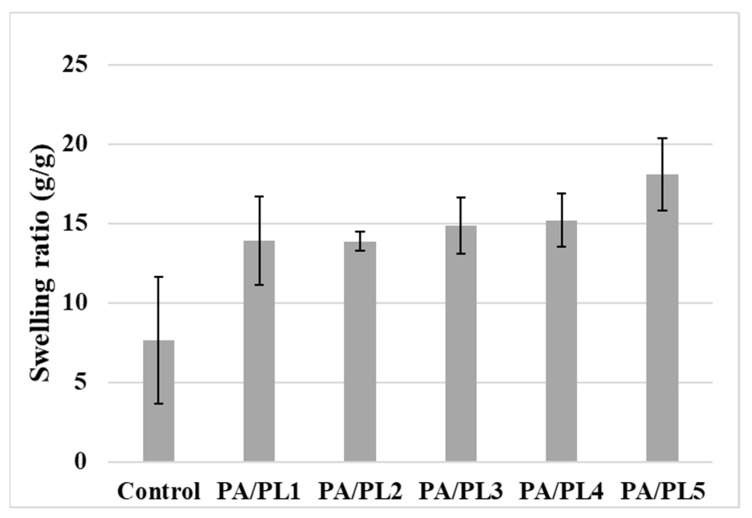
Average swelling ratio of nanofiber membranes.

The hydrophilic behavior of the samples was further analyzed according to water absorption capacity by measuring the swelling ratio (see Table 4 and Figure 5). The swelling ratio of samples increased with PL concentration, with a significant increase for PA/PL5 with respect to the control sample. The increased swelling ratio indicates better water uptake capacity through available hydrophilic sites [36,37]. These results are in alignment with the water contact angle results; therefore, it can be said that hydrophilicity of samples improved with the increase in PL concentration owing to its inherent hydrophilic tendency [17,19,32]. The improved hydrophilic behavior of PA due to PL can improve its user comfort along with its applicability in medical textiles such as wipes, wound dressings, and the inner layer of face masks, where hydrophilicity is desirable [32,33].

### 3.5. Thermal Analysis

**Figure 6:**
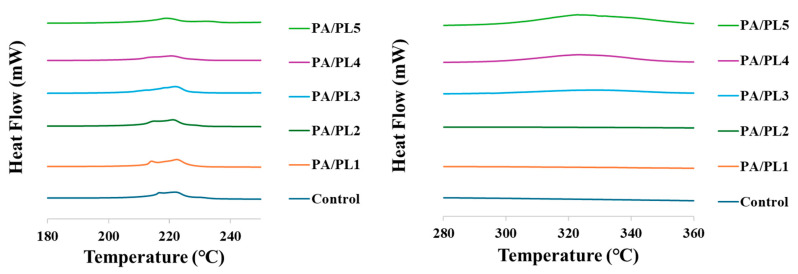
Differential scanning calorimetry curve of nanofiber membranes.

The thermal analyses of nanofiber membranes are shown in Figure 6. Endothermic peaks were observed for all the samples, representing the melting behavior of the nanofiber membranes. The thermal analysis of PA/PL3, PA/PL4, and PA/PL5 samples showcased multiple melting peaks indicating secondary crystallization (melting–recrystallization–remelting) [49,50]. The onset of melting for control, PA/PL1, PA/PL2, PA/PL3, PA/PL4, and PA/PL 5 was at 207.05 °C, 207.54 °C, 207.01 °C, 207.78 °C, 208.17 °C, and 208.3 °C, respectively. The improved thermal stability of the nanofiber membranes upon addition of PL indicates no disruption to the PA structure and a possible interaction between PA and PL polymers [18].

### 3.6. Mechanical Analysis

The mechanical properties of the samples in terms of tensile strength and elongation are summarized in Table 4. The addition of PL indicated an increasing trend in the tensile strength of PA, with a significant increase for PA/PL5 from the control sample. The increasing tensile strength is an indication of no disruption to the PA structure after the addition of PL, along with a possible interaction between the polymers [51]. The increasing tensile strength could be tied to the FTIR analysis, suggesting hydrogen bonding upon addition of PL, as the introduction of amino and amide bonds to the peptide chain provides more hydrogen bonding [47]. Further, the improved thermal stability, with increasing concentration of PL, as shown in thermal analysis, also indicates an interaction between the two polymers [18]. As for the elongation, the initial PL concentrations indicated an increasing trend in the elongation (%). However, as expected, the increase in elongation declined for higher PL concentration. The reduced elongation (%) with increased tensile strength can be tied to the increased rigidity and crystallinity as a result of interactions between PA and PL. However, the elongation (%) did not present a significant deterioration with respect to the control sample.

### 3.7. Antimicrobial Analysis

**Figure 7:**
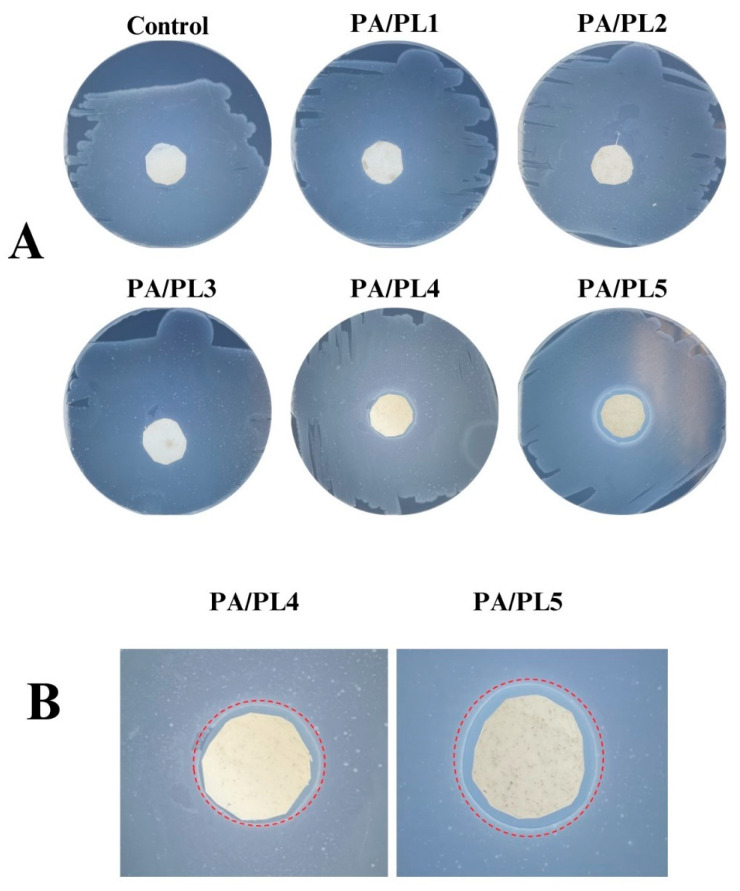
(**A**) Antimicrobial activity of the nanofiber membranes and (**B**) zoomed-in views of the observed zones of inhibition.

**Figure 8:**
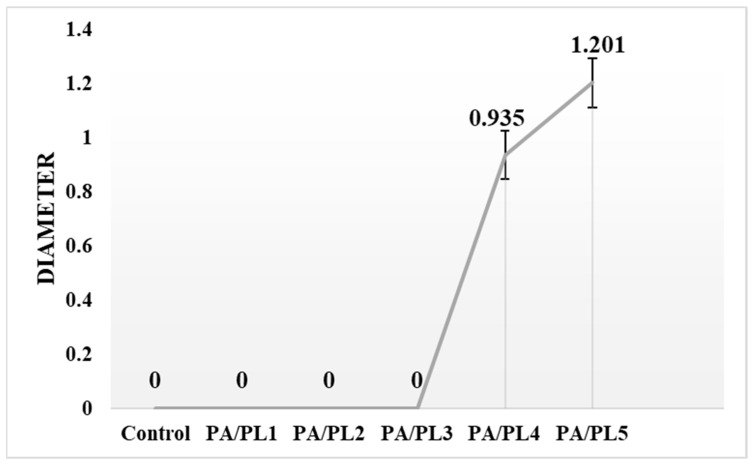
Average areas of antimicrobial zones of inhibition for all the nanofiber membranes.

The antimicrobial activity of the developed compositions was tested through zone inhibition testing. The testing demonstrates the antimicrobial function of the test sample upon the observation of an inhibition zone surrounding the sample when plated with test bacteria [52]. Figure 7 is a representative image of the testing. Zones of inhibition were seen for the samples PA/PL4 and PA/PL5, indicating antimicrobial activity against the tested bacteria *E. coli*, with highest antimicrobial activity for PA/PL5 (see Figure 8). PL has an inherent cationic antimicrobial function owing to the α-amino side group [12,14,15]. The FTIR analysis confirmed the presence of PL and a cationic amino group in the samples, which are responsible for inducing antimicrobial activity to base PA. The antimicrobial tests disclosed that base PA consisting of PL at 40% (*w*/*w*) of PA is successful in establishing efficient antimicrobial function. The addition of PL in concentrations lower than 25% (*w*/*w*) of PA did not exhibit an inhibition zone and are thus insufficient to induce antimicrobial function to the base PA. The efficient antimicrobial activity of PA/PL5 could be tied to a high concentration of PL and defect-free homogeneous morphology.

### 3.8. Long-Term Stability of Antimicrobial Property

**Figure 9:**
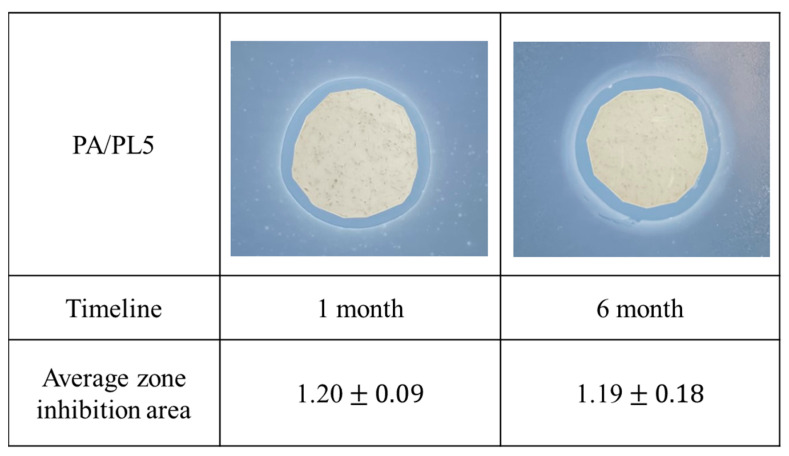
Long-term stability of antimicrobial activity for PA/PL5.

The characterization of an antimicrobial agent in textiles can be versatile, addressing aspects crucial to its intended applications, such as storage, reusability, or long-term stability [9,53,54]. PL is a cationic polymer exhibiting an antimicrobial function through the positively charged amino side groups. Although cationic polymers such as PL have the advantage of better stability over the leaching antimicrobial agent, cationic polymers can still experience a proliferation of bacteria on the surface once the surface is sufficiently covered with microorganisms due to the exhaustion of cations [7,23,24,53]. Thus, it is important to analyze the long-term stability of antimicrobial textiles. Despite the importance of this test to being able to define an appropriate shelf-life, ensuring functionality, its inclusion in antimicrobial characterization was found to be uncommon [27]. The long-term stability of the PA/PL 5 sample was characterized at 1 and 6 months. As seen in Figure 9, the antimicrobial activity through the observed zone of inhibition remains to be effective until 6 months. This test allows us to define the 6-month shelf-life of developed PA/PL5 without any deterioration in antimicrobial activity. 

## 4. Conclusions and Future Scope

PA is a popular polymer for functional textile applications due to its excellent mechanical and thermal properties, chemical stability, and biocompatibility. However, PA nanofibers tend to promote bacterial growth, limiting their use in medical textiles. On the other hand, PL is a naturally occurring homopolyamide known for its non-toxicity, hydrophilicity, and antimicrobial properties, but its low molecular weight hinders its spinnability. Given the structural similarity between PL and PA, with PL having an additional amino side chain, this study addresses the spinnability of PL by utilizing PA as the carrier, hypothesizing enhanced PA properties.

This study demonstrates the spinnability of varying concentrations of PL with base PA, including higher concentration of PL than previously reported. The effects of PL addition on morphology, hydrophilicity, thermal stability, mechanical property, and antimicrobial activity were thoroughly investigated. Among the various compositions developed and characterized, PA/PL5 was identified as the maximum spinnable concentration of PL that allowed for enhanced properties in PA, resulting in superhydrophilicity, improved thermal and mechanical behavior, and efficient antimicrobial properties with long-term stability.

This study presents a simple scalable method with which to produce spinnable fibers with PA backbone and the added cationic α-amino group from PL. This method can enhance the industrial utilization of PA in functional textiles by improving mechanical performance, improving hydrophilicity, and providing stable antimicrobial function. These findings suggest significant potential for specific applications, such as respiratory protection, wound dressing, and sanitation.

## Figures and Tables

**Table 1:** Spinning solution compositions.

Sample Code	Polyamide-6 Concentration	ε-Poly-L-lysine Concentration
Control	30% (*w*/*v*) of formic acid	0
PA/PL1	30% (*w*/*v*) of formic acid	0.5% (*w*/*w*) of PA
PA/PL2	30% (*w*/*v*) of formic acid	1.33% (*w*/*w*) of PA
PA/PL3	30% (*w*/*v*) of formic acid	10% (*w*/*w*) of PA
PA/PL4	30% (*w*/*v*) of formic acid	25% (*w*/*w*) of PA
PA/PL5	30% (*w*/*v*) of formic acid	40% (*w*/*w*) of PA
PA/PL6	30% (*w*/*v*) of formic acid	65% (*w*/*w*) of PA
PA/PL7	15% (*w*/*v*) of formic acid	100% (*w*/*w*) of PA

**Table 2:** Electrospinning parameters.

Sample	Feed Rate	Supply Voltage	Spinning Distance	Collection Time
Control and PA/PL1 to PA/PL6	1 mL/h	20 KV	12 cm	4 h
PA/PL7_A	1 mL/h	18 KV	12 cm	4 h
PA/PL7_B	1 mL/h	16 KV	12 cm	4 h
PA/PL7_C	1 mL/h	16 KV	14 cm	4 h

**Table 3:** Average diameter of nanofibers.

Sample Code	Diameter (Microns)
Control	0.22 ± 0.14 ^ab^
PA/PL1	0.18 ± 0.10 ^ab^
PA/PL2	0.16 ± 0.05 ^b^
PA/PL3	0.18 ± 0.05 ^ab^
PA/PL4	0.21 ± 0.05 ^ab^
PA/PL5	0.43 ± 0.07 ^c^
PA/PL6	1.06 ± 0.50 ^d^
PA/PL7_A	0.24 ± 0.05 ^ab^
PA/PL7_B	0.26 ± 0.07 ^ab^
PA/PL7_C	0.28 ± 0.12 ^a^

Different letters in the superscripts indicate significant difference (*p* < 0.05).

**Table 4:** Hydration and mechanical properties of nanofiber membranes.

Sample Code	Average Static Water Contact Angle	Average Swelling Ratio (g/g)	Tensile Strength (MPa)	Elongation (%)
Control	82.44 ± 9.72 ^a^	7.63 ± 3.98 ^a^	0.36 ± 0.03 ^a^	3.00 ± 0.84 ^a^
PA/PL1	40.44 ± 1.59 ^b^	13.92 ± 2.79 ^ab^	0.33 ± 0.21 ^a^	7.83 ± 3.85 ^b^
PA/PL2	18.25 ± 4.02 ^c^	13.87 ± 0.61 ^ab^	0.53 ± 0.05 ^a^	6.46 ± 1.19 ^ab^
PA/PL3	0 ^d^	14.86 ± 1.75 ^ab^	0.72 ± 0.26 ^ab^	5.60 ± 1.01 ^ab^
PA/PL4	0 ^d^	15.20 ± 1.69 ^ab^	0.75 ± 0.19 ^ab^	5.13 ± 0.40 ^ab^
PA/PL5	0 ^d^	18.10 ± 2.29 ^b^	1.02 ± 0.25 ^b^	2.33 ± 0.50 ^a^

Different letters in the superscripts indicate significant difference (*p* < 0.05).

## Data Availability

The original contributions presented in the study are included in the article, further inquiries can be directed to the corresponding authors.

## References

[1] Zhang X. (2014). Fundamentals of Fiber Science.

[2] Xue J., Wu T., Dai Y., Xia Y. (2019). Electrospinning and electrospun nanofibers: Methods, materials, and applications. Chem. Rev..

[3] Khan W.S., Asmatulu R., Ceylan M., Jabbarnia A. (2013). Recent progress on conventional and non-conventional electrospinning processes. Fibers Polym..

[4] Li Y., Zhu J., Cheng H., Li G., Cho H., Jiang M., Gao Q., Zhang X. (2021). Developments of advanced electrospinning techniques: A critical review. Adv. Mater. Technol..

[5] Gupta D. (2011). Functional clothing-Definition and classification. Indian J. Fiber Text. Res..

[6] Zhang Y., Xia X., Ma K., Xia G., Wu M., Cheung Y.H., Yu H., Zou B., Zhang X., Farha O.K. (2023). Functional Textiles with Smart Properties: Their Fabrications and Sustainable Applications. Adv. Funct. Mater..

[7] Riga E.K., Vöhringer M., Widyaya V.T., Lienkamp K. (2017). Polymer-based surfaces designed to reduce biofilm formation: From antimicrobial polymers to strategies for long-term applications. Macromol. Rapid Commun..

[8] Qiu H., Si Z., Luo Y., Feng P., Wu X., Hou W., Zhu Y., Chan-Park M.B., Xu L., Huang D. (2020). The mechanisms and the applications of antibacterial polymers in surface modification on medical devices. Front. Bioeng. Biotechnol..

[9] Stokes K., Peltrini R., Bracale U., Trombetta M., Pecchia L., Basoli F. (2021). Enhanced medical and community face masks with antimicrobial properties: A systematic review. J. Clin. Med..

[10] Bhandari V., Jose S., Badanayak P., Sankaran A., Anandan V. (2022). Antimicrobial finishing of metals, metal oxides, and metal composites on textiles: A systematic review. Ind. Eng. Chem. Res..

[11] Karypidis M., Karanikas E., Papadaki A., Andriotis E.G. (2023). A Mini-Review of Synthetic Organic and Nanoparticle Antimicrobial Agents for Coatings in Textile Applications. Coatings.

[12] Tao Y., Chen X., Jia F., Wang S., Xiao C., Cui F., Li Y., Bian Z., Chen X., Wang X. (2015). New chemosynthetic route to linear ε-poly-lysine. Chem. Sci..

[13] Patil N.A., Kandasubramanian B. (2021). Functionalized polylysine biomaterials for advanced medical applications: A review. Eur. Polym. J..

[14] Shoji S., Hiroyoshi M., Toshiro I., Heiichi S. (1984). Antimicrobial action of ε-poly-l-lysine. Jpn. J. Antibiot..

[15] Li M., Tao Y. (2021). Poly (ε-lysine) and its derivatives via ring-opening polymerization of biorenewable cyclic lysine. Polym. Chem..

[16] Lin L., Xue L., Duraiarasan S., Haiying C. (2018). Preparation of ε-polylysine/chitosan nanofibers for food packaging against Salmonella on chicken. Food Packag. Shelf Life.

[17] Wu C., Sun J., Lu Y., Wu T., Pang J., Hu Y. (2019). In situ self-assembly chitosan/ε-polylysine bionanocomposite film with enhanced antimicrobial properties for food packaging. Int. J. Biol. Macromol..

[18] Liu F., Liu Y., Sun Z., Wang D., Wu H., Du L., Wang D. (2020). Preparation and antibacterial properties of ε-polylysine-containing gelatin/chitosan nanofiber films. Int. J. Biol. Macromol..

[19] Mayandi V., Wen Choong A.C., Dhand C., Lim F.P., Aung T.T., Sriram H., Dwivedi N., Periayah M.H., Sridhar S., Fazil M.H. (2020). Multifunctional antimicrobial nanofiber dressings containing ε-polylysine for the eradication of bacterial bioburden and promotion of wound healing in critically colonized wounds. ACS Appl. Mater. Interfaces.

[20] Dias Y.J., Robles J.R., Sinha-Ray S., Abiade J., Pourdeyhimi B., Niemczyk-Soczynska B., Kolbuk D., Sajkiewicz P., Yarin A.L. (2021). Solution-blown poly (hydroxybutyrate) and ε-poly-l-lysine submicro-and microfiber-based sustainable nonwovens with antimicrobial activity for single-use applications. ACS Biomater. Sci. Eng..

[21] Zhang X., Shi C., Liu Z., Pan F., Meng R., Bu X., Xing H., Deng Y., Guo N., Yu L. (2018). Antibacterial activity and mode of action of ε-polylysine against *Escherichia coli* O157: H7. J. Med. Microbiol..

[22] Wahid F., Wang F.P., Xie Y.Y., Chu L.Q., Jia S.R., Duan Y.X., Zhang L., Zhong C. (2019). Reusable ternary PVA films containing bacterial cellulose fibers and ε-polylysine with improved mechanical and antibacterial properties. Colloids Surf. B Biointerfaces.

[23] Banerjee I., Pangule R.C., Kane R.S. (2011). Antifouling coatings: Recent developments in the design of surfaces that prevent fouling by proteins, bacteria, and marine organisms. Adv. Mater..

[24] Babutan I., Lucaci A.D., Botiz I. (2021). Antimicrobial polymeric structures assembled on surfaces. Polymers.

[25] Matulevicius J., Kliucininkas L., Martuzevicius D., Krugly E., Tichonovas M., Baltrusaitis J. (2014). Design and characterization of electrospun polyamide nanofiber media for air filtration applications. J. Nanomater..

[26] Dinç S.D., Göktepe F. (2021). Production and analysis of electrospun PA 6, 6 and PVA nanofibrous surfaces for filtration. Ind. Textila.

[27] Lencova S., Zdenkova K., Jencova V., Demnerova K., Zemanova K., Kolackova R., Hozdova K., Stiborova H. (2021). Benefits of polyamide nanofibrous materials: Antibacterial activity and retention ability for Staphylococcus aureus. Nanomaterials.

[28] Lencova S., Svarcova V., Stiborova H., Demnerova K., Jencova V., Hozdova K., Zdenkova K. (2020). Bacterial biofilms on polyamide nanofibers: Factors influencing biofilm formation and evaluation. ACS Appl. Mater. Interfaces.

[29] Lencova S., Stiborova H., Munzarova M., Demnerova K., Zdenkova K. (2022). Potential of Polyamide Nanofibers With Natamycin, Rosemary Extract, and Green Tea Extract in Active Food Packaging Development: Interactions With Food Pathogens and Assessment of Microbial Risks Elimination. Front. Microbiol..

[30] Ushimaru K., Hamano Y., Katano H. (2017). Antimicrobial activity of ε-poly-L-lysine after forming a water-insoluble complex with an anionic surfactant. Biomacromolecules.

[31] Ushimaru K., Morita T., Fukuoka T. (2020). Bio-based, flexible, and tough material derived from ε-Poly-l-lysine and fructose via the maillard reaction. ACS Omega.

[32] Songer J. Adopting Who Guidance on Fabric Masks for COVID-19. https://papers.ssrn.com/sol3/papers.cfm?abstract_id=3632531.

[33] Zhang W., Li J.X., Tang R.C., Zhai A.D. (2020). Hydrophilic and antibacterial surface functionalization of polyamide fabric by coating with polylysine biomolecule. Prog. Org. Coat..

[34] Ojha S.S., Afshari M., Kotek R., Gorga R.E. (2008). Morphology of electrospun nylon-6 nanofibers as a function of molecular weight and processing parameters. J. Appl. Polym. Sci..

[35] (2022). Standard Practice for Surface Wettability of Coatings, Substrates and Pigments by Advancing Contact Angle Measurement.

[36] Almasian A., Giahi M., Fard G.C., Dehdast S.A., Maleknia L. (2018). Removal of heavy metal ions by modified PAN/PANI-nylon core-shell nanofibers membrane: Filtration performance, antifouling and regeneration behavior. Chem. Eng. J..

[37] Cay A., Miraftab M. (2013). Properties of electrospun poly (vinyl alcohol) hydrogel nanofibers crosslinked with 1, 2, 3, 4-butanetetracarboxylic acid. J. Appl. Polym. Sci..

[38] (2009). Standard Test Method for Tensile Properties of Thin Plastic Sheeting.

[39] (2011). Test Method for Antibacterial Activity of Textile Materials: Parallel Streak Method.

[40] Koombhongse S., Liu W., Reneker D.H. (2001). Flat polymer ribbons and other shapes by electrospinning. J. Polym. Sci. Part B Polym. Phys..

[41] Itoh H., Li Y., Chan K.H., Kotaki M. (2016). Morphology and mechanical properties of PVA nanofibers spun by free surface electrospinning. Polym. Bull..

[42] Şener A.G., Altay A.S., Altay F. (2011). Effect of voltage on morphology of electrospun nanofibers. Proceedings of the 2011 7th International Conference on Electrical and Electronics Engineering (ELECO).

[43] Haider A., Haider S., Kang I.K. (2018). A comprehensive review summarizing the effect of electrospinning parameters and potential applications of nanofibers in biomedical and biotechnology. Arab. J. Chem..

[44] Han Y., Shi C., Cui F., Chen Q., Tao Y., Li Y. (2020). Solution properties and electrospinning of polyacrylamide and ε-polylysine complexes. Polymer.

[45] Bhardwaj N., Kundu S.C. (2010). Electrospinning: A fascinating fiber fabrication technique. Biotechnol. Adv..

[46] Hsu C.P. (1997). Infrared spectroscopy. Handbook of Instrumental Techniques for Analytical Chemistry.

[47] Zhang S., Chen H., Shi Z., Liu Y., Liu L., Yu J., Fan Y. (2023). Preparation of amino cellulose nanofiber via ε-poly-L-lysine grafting with enhanced mechanical, anti-microbial and food preservation performance. Ind. Crops Product..

[48] Rotter G., Ishida H. (1992). FTIR separation of nylon-6 chain conformations: Clarification of the mesomorphous and γ-crystalline phases. J. Polym. Sci. Part B Polym. Phys..

[49] Gunaratne L.M., Shanks R.A. (2005). Multiple melting behaviour of poly (3-hydroxybutyrate-co-hydroxyvalerate) using step-scan DSC. Eur. Polym. J..

[50] Asran A.S., Razghandi K., Aggarwal N., Michler G.H., Groth T. (2010). Nanofibers from blends of polyvinyl alcohol and polyhydroxy butyrate as potential scaffold material for tissue engineering of skin. Biomacromolecules.

[51] Han Y., Xu Y., Zhang S., Li T., Ramakrishna S., Liu Y. (2020). Progress of improving mechanical strength of electrospun nanofibrous membranes. Macromol. Mater. Eng..

[52] Pinho E., Magalhães L., Henriques M., Oliveira R. (2011). Antimicrobial activity assessment of textiles: Standard methods comparison. Ann. Microbiol..

[53] Tofail S.A., Bauer J. (2016). Electrically Mediated Interactions at the Materials/Biology Interface. Electrically Active Materials for Medical Devices.

[54] Monticello R.A., Askew P.D. (2013). Antimicrobial textiles and testing techniques. Russell, Hugo & Ayliffe’s: Principles and Practice of Disinfection, Preservation and Sterilization.

